# Comparison of the number of live births, maternal age at childbirth, and weight of live births between Korean women and immigrant women in 2018

**DOI:** 10.4069/kjwhn.2021.03.15

**Published:** 2021-03-23

**Authors:** Sun-Hee Kim, Sooyoung Kim, Byeongje Park, Seokmin Lee, Sanghee Park, Geum Hee Jeong, Kyung Won Kim, Sook Jung Kang

**Affiliations:** 1 College of Nursing, Daegu Catholic University, Daegu, Korea; 2 Vital Statistics Division, Statistics Korea, Daejeon, Korea; 3 School of Nursing and Research Institute in Nursing Science, Hallym University, Chuncheon, Korea; 4 Department of Nursing, Daegu Haany University, Daegu, Korea; 5 College of Nursing, Ewha Womans University, Seoul, Korea

**Keywords:** Birth weight, Emigrants and immigrants, Gestational age, Live birth, Newborn infant

## Abstract

**Purpose:**

This study compared maternal age at childbirth, the number of live births, and the weight of live births between Korean women and immigrant women using statistical data from the Republic of Korea for the period of 2008-2018.

**Methods:**

The analysis was conducted using data from the Microdata Integrated Service of Statistics Korea (https://mdis.kostat.go.kr/index.do).

**Results:**

Korean women and immigrant women showed a higher age at childbirth in 2018 than in 2008. The percentage of newborns of Korean women with a birth weight of less than 2.5 kg increased slightly for 3 consecutive years from 2016 to 2018, whereas for immigrant women, this percentage increased in 2017 compared to 2016 and then decreased again in 2018. Very low birth weight (less than 1.5 kg) became more common among immigrant women from 2016 to 2018. Birth at a gestational age of fewer than 37 weeks increased both among Korean and immigrant women from 2016 to 2018. In both groups, the percentage of women who had their first child within their first 2 years of marriage decreased from 2008 to 2018.

**Conclusion:**

Immigrant women had higher birth rates than Korean women, while both groups showed an increasing trend in preterm birth. Greater attention should be paid to the pregnancy and birth needs of immigrant women, and steps are needed to ensure health equity and access in order to prevent preterm births. It is also necessary to identify factors that affect preterm birth and birth of very low birth weight infants among immigrant women in the future.

## Introduction

### Background and rationale

The total fertility rate in the Republic of Korea (hereafter, Korea) fell below the population replacement level of 2.0 in 1985 and steadily decreased to below 1.0, reaching as low as 0.92 in 2019 [1]. This trend is reflected in the decrease of number of live births from 465,892 in 2008 to 302,676 in 2019 [1]. Those two tendencies are closely related to decreasing trends in the frequency of marriages in Korea and the late marriage phenomenon. Additionally, the crude marriage rate of Korean women decreased from 10.6 in 1980 to 4.7 in 2019 [1]. In 2019, women married at an average age of 30.59 years [2] and on average gave birth for the first time at age of 32.2 years [3]. The age at which a woman gives birth for the first time influences the health of the child. For instance, the rate of successful implantation of a fertilized ovum is reduced after 35 years of age. Women over the age of 35 years struggle to maintain pregnancy and face increased risks of congenital disabilities and fetal and maternal complications during pregnancy [4]. Therefore, such women are often unwilling to conceive second and third children due to the difficulties of raising children and the risk of pregnancy at an advanced maternal age. For this reason, the low birth rate in Korean society is expected to accelerate if women in Korea do not start bearing children at a younger age.

Despite the low birth rate in Korea, international marriages have become more widespread, and marriages between Korean men and foreign women accounted for 6.17% of marriages, on average, from 2010 to 2019 [5,6]. In 2019, the average age of immigrant women at marriage was 28.4 years [7], and immigrant women birthed their first child at an average age of 29.6 years [8]. Considering that Korean women gave birth to their first child at an average age older than 32 years in 2019 [3], immigrant women bear children at a younger age than Korean women. As a result, the health and social outcomes of immigrant women will have cultural and social effects on Korea. Another important consideration is health equity for immigrant women, as they have the right to receive childbirth-related care even though their nationality is or was not Korean.

In 2019, 5.90% of live births in Korea occurred among immigrant women [8]. Although the Multicultural Families Support Act was established in 2008 and three phases of multicultural family policy have been administered by the government since 2008, policies related to reproductive health continue to be lacking [9]. As a result, immigrant women face several difficulties in pregnancy and childbirth. First, immigrant women often experience pregnancy and childbirth before they can adapt to Korean society [10]. Furthermore, immigrant women rarely visit medical institutions due to a lack of medical insurance, high health insurance costs, or difficulties in visiting [11,12]. In fact, only 31.6% of immigrant women were found to have received pregnancy and childbirth support services [13]. Additionally, some immigrant women do not receive sufficient information about pregnancy and childbirth and do not have the opportunity to manage prenatal care [10-12]. Improper prenatal care affects women’s ability to maintain pregnancy, increases the chance of pregnancy complications, and impacts babies’ health [14]. Previous studies did not compare data related to childbirth between Korean women and immigrant women on the basis of complete survey data; instead, comparisons were made only by reasoning about non-representative data. A comparison of childbirth statistics among Korean women and immigrant women may reveal more accurate data related to childbirth among immigrant women, as well as hidden disparities, with potential implications for predicting the health of babies born to immigrant women. This comparison will help future researchers create interventional measures and policies for immigrant women’s childbirth and health.

### Objectives

The purpose of this study was to compare the number of live births, the weight and gestational period of birth, maternal age at childbirth, and the period from marriage to the first childbirth between Korean women and immigrant women using census microdata of Statistics Korea. Ultimately, the study aimed to provide suggestions to promote safe and healthy pregnancy and birth among immigrant women by providing basic data for clinical practice and policy preparations. The specific objectives of this study were to compare the following four specific parameters between Korean and immigrant women: (1) the number of live births, (2) the number of live births by maternal age, (3) the weights of live birth and gestational age, and (4) the husband’s age and length of marriage at the first childbirth.

## Methods

Ethics statement: This study was a secondary analysis of existing data and did not require institutional review board approval or informed consent.

### Study design

This study was a chronological analytic study based on birth population data.

### Data sources

The researchers used the 2008 to 2018 Census of Population Dynamics data from the Microdata Integrated Service (MDIS) provided by Statistics Korea [15]. Statistics Korea produces statistics on the population dynamics of births, deaths, marriages, and divorces, which are complete survey data (i.e., not sample survey data). Statistics Korea uses birth certificates and birth declaration forms to formulate statistics. For example, when a baby is born, a birth certificate issued by the health care provider is submitted to a local governance organization. The birth certificate includes information such as place of birth, gestational age, information on multiple fetuses, birth weight, and birth height. The birth report includes information on whether a person is married, the educational background of the parents, parents’ age, and parents’ nationality. The criteria for immigrant women in this study were limited to naturalized women or foreign nationals, and the researchers excluded cases of women with an unknown nationality from the analysis. The researchers used data from the MDIS, which contains nationality data of immigrant women [15], to analyze the 2008 to 2018 birth data.

### Study variables

The study variables were as follows; (1) the number of live births and its composition ratio of Korean women and immigrant women, (2) the number and composition ratio of births by the age of Korean women and immigrant women, (3) the nationality of immigrant women who gave birth, (4) the average age at childbirth, (5) the average age of husbands at childbirth, (6) length of marriage at the first childbirth, (7) the number of live births according to the nationality of immigrant women, (8) the number of live births according to gestational age, and (9) birth weight.

### Definition of terms used in this study

In the context of this study, the term “Korean women” refers to women who were born in Korea or women born abroad with Korean nationality. The term “immigrant women” refers to women who gave birth as either naturalized Koreans or foreign nationals. The term “live birth” refers to “the complete expulsion or extraction of a product of human conception from its mother irrespective of the duration of pregnancy, which—after such expulsion or extraction—breathes or shows any other evidence of life such as beating of the heart, pulsation of the umbilical cord, or definite movement of voluntary muscles, whether or not the umbilical cord has been cut or the placenta is attached.” Heartbeats are to be distinguished from transient cardiac contractions, and respirations are to be distinguished from fleeting respiratory efforts or gasps [16] .

### Statistical methods

The researchers completed comparative analyses and observational statistics. Since the data are complete survey data, descriptive statistics including frequency and percentage were used for the comparison. The researchers used IBM SPSS ver. 23.0 for Windows (IBM Corp., Armonk, NY, USA) for the statistical analysis.

## Results

### Comparison of live births between Korean women and immigrant women

**Figure 1.**
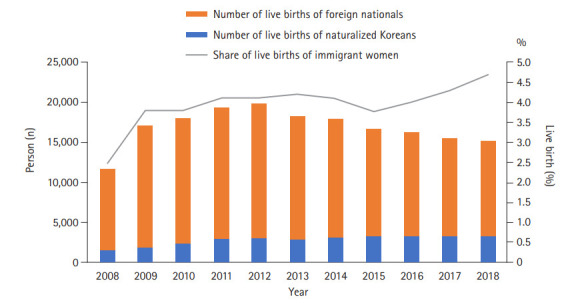
Number of live births of immigrant women in 2008-2018 in Korea.

The number of live births in Korea steadily decreased from 465,892 in 2008 to 326,822 in 2018, representing a 29.9% reduction (Supplementary Table 1). The number and percentage of births of Korean women declined from 2008 (n=451,376, 96.9%) to 2018 (n=311,418, 95.3%), whereas the number and percentage of births in immigrant women steadily increased from 2008 (n=11,690, 2.5%) to 2018 (n=15,216, 4.7%) (Figure 1 and Supplementary Table 1).

Two-thirds of immigrant women (n=10,157, 66.7%) were from Vietnam (n=6,411, 42.1%) and China (n=3,746, 24.6%), followed by women from the Philippines (n=1,243, 8.2%) and Cambodia (n=736, 4.8%) (Table 1).

### Comparison of live births by age between Korean women and immigrant women

**Figure 2.**
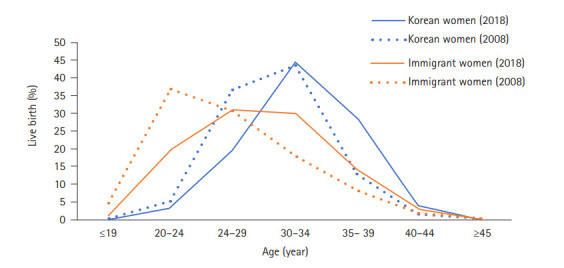
Percentage of the number of live births by age of Korean women and immigrant women in 2008 and 2018.

Figure 2 and Supplementary Table 2 display a comparison of the number of live births by age between Korean women and immigrant women. Compared to 2008, the percentages of births in both younger Korean and immigrant women decreased in 2018, and the percentage of older women increased in 2018 (Figure 2). Only 23.1% of Korean women (n=71,726) were younger than 30 years at childbirth in 2018, whereas this was the case for 52.0% of immigrant women (n=7,913). Almost one-third of Korean women (n =101,329, 32.5%) were 35 years old or older at childbirth in 2018, whereas only 17.1% of immigrant women (n=2,596) were 35 years or older at the time of childbirth.

### Comparison of birth weight and gestational age between Korean women and immigrant women

Among Korean women, the percentage of low birth weight (LBW) newborns (<2.5 kg) increased from 2016 (5.9%) to 2018 (6.3%); however, among immigrant women, the proportion of LBW newborns increased from 2016 (5.3%) to 2017 (6.0%) and then decreased again in 2018 (5.6%). Although the percentage of very low birth weight (VLBW) newborns (<1.5 kg) who were born to Korean women was similar from 2016 (0.7%) to 2018 (0.7%), the percentage of VLBW newborns in immigrant women steadily increased from 2016 (0.6%) to 2018 (0.8%). Among Korean women, the percentage of newborns with a birth weight of 4.0 kg and higher decreased from 2016 (3.3%) to 2018 (2.9%); however, the percentage of newborns in the same weight range who were born to immigrant women remained almost unchanged (Table 2).

The number of preterm births (i.e., newborns born before 37 weeks of gestation) increased from 2016 to 2018 among both Korean and immigrant women. This is applied to both singleton and multifetal pregnancies.

### Comparisons of the age of Korean women, immigrant women, and their husbands at childbirth and length of marriage at the first childbirth

**Figure 3.**
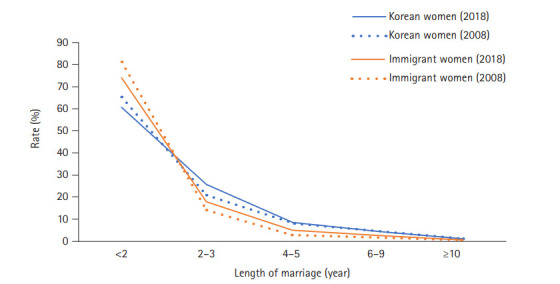
Distribution of length of marriage at the first childbirth in 2008 and 2018.

Supplementary Table 3 shows the average age of women and their husbands when they had their first, second, and third or later newborn. As expected, immigrant women were younger (28.2 years) than Korean women (31.6 years) when giving birth to their first child. Unsurprisingly, the husbands of immigrant women who were expecting their first child were typically older (39.6 years) than the husbands of Korean women (33.8 years). In 2018, the percentage of immigrant women who had their first newborn within their first 2 years of marriage (n=5,185, 65.4%) was higher than the corresponding percentage of Korean women (n=99,644, 60.3%). However, the percentage of immigrant women who had their first newborn within 2 to 3 years after marriage (n=1,676, 21.1%) was lower than the corresponding percentage of Korean women (n=43,126, 26.1%) (Supplementary Table 3 and Figure 3). For both Korean and immigrant women, the percentage of women who had their first child within the first 2 years of marriage decreased in 2018 compared to 2008. In contrast, the percentage women who had their first child after more than 2 years of marriage increased in 2018 among both Korean and immigrant women (Supplementary Table 3 and Figure 3).

## Discussion

Although the number of babies born in Korea has decreased, the proportion of live births by immigrant women has increased. The age at childbirth among immigrant women is mostly from 20 to 35 years. The rate of high-risk births, such as preterm births and VLBW newborns (less than 1.5 kg) also increased among immigrant women.

The number of live births among Korean women has continued to decline, and the number of babies born to immigrant women also decreased since 2012 (Supplementary Table 2); however, the rate of this decrease was lower among immigrant women than among Korean women. Thus, these findings reflect an increase in the proportion of live births among immigrant women out of all live births in Korea. This increase in the proportion of live births among immigrant women in Korea implies that Korea will soon become a multicultural society, with people of non-Korean origin accounting for over 5% of the population; in fact, this change is nearly reality, as 4.9% of Korea’s population was composed of people of non-Korean origin in 2019 [17]. The increase in the number of live births among immigrant women has contributed to the quantitative increase of the Korean population; however, previous studies reported that immigrant women struggled to manage their pregnancy and childbirth-related health. Immigrant women are not adequately informed about pregnancy and childbirth due to difficulties in communication, an inability to adapt to unfamiliar hospital systems, and frequent experiences of discrimination during pregnancy and childbirth [11,12].

Korea has a high number of Vietnamese and Chinese immigrant women; thus, Korean healthcare providers should offer more educational materials and pregnancy and childbirth programs for this population [18]. While efforts have been made to assist Cambodian immigrant women to conceive, give birth, and adapt to Korean society [19], Cambodian immigrant women account for only 2.5% of all immigrant women and 4.8% of live births among all immigrant women in Korea. Therefore, it is necessary to improve the quality of pregnancy and childbirth management of all immigrant women by facilitating immigrant women’s adaptation to the Korean medical system and establishing measures to ensure proper communication with medical personnel [11]. These steps will be possible through policy support within the medical system. The third phase of the multicultural family policy focuses on social and economic participation as well as support of children’s growth [9]. Such policy and actual programs also need to be developed and applied in both community and health care settings to have meaningful outcomes. Researchers must also identify the specific needs and cultural characteristics of pregnancy and childbirth of immigrant women according to their country of origin. Researchers and healthcare providers must further develop pregnancy and childbirth education materials and strengthen support services for immigrant women to promote health equity and ensure a healthy next generation of Koreans.

The study results revealed that the proportion of VLBW newborns who were born to immigrant women increased steadily from 2016 to 2018. Furthermore, the birth rate of infants born under 37 weeks of gestation increased from 2016 to 2018, especially among immigrant women. Additionally, the preterm birth rate of singletons born to immigrant women has continued to increase in comparison to Korean women. Infants born to immigrant women at university hospitals had an average gestation period of 35 weeks, and the majority of children were underweight (less than 2.5 kg) [14]. International reports, according to which immigrant women often give birth to underweight babies [11,20], suggest that it is not easy for immigrant women to manage pregnancy and childbirth due to language and cultural differences. Immigrant women are often within a low socioeconomic bracket, have difficulties accessing medical facilities, and are at risk for physical abuse, cultural maladjustment, and exposure to various preterm birth-related risk factors such as preconception malnutrition and pregnancy-related diseases [21].

Furthermore, VLBW babies often suffer from neurological disorders and have a high mortality rate [22]. Mothers of LBW newborns can experience distress [23] and child-rearing stress [24]; thus, women of low-weight babies often need child-rearing support. Convenient access to the medical system will (1) allow all immigrant women to receive prenatal care, (2) reduce the birth rate of underweight infants, and (3) increase the live birth rate after 37 weeks of gestation. Healthcare providers who identify the risk factors of childbirth by nationality and devise interventions that correspond to immigrant women’s cultural characteristics can improve the quality of pregnancy and childbirth among immigrant women.

In 2018, 81.6% of immigrant women gave birth between the ages of 20 and 35 years and were consequently at a lower risk for pregnancy complications involving both the mother and fetus. Among immigrant women, the period of time between the date of marriage and the birth of their first child increased by more than 2 years from 2008 to 2018. However, in 2018, 65.4% of immigrant women still gave birth to their first baby within less than 2 years of marriage, meaning that these women had not adapted to Korean society before birthing their first child. Immigrant women must adapt to daily life during pregnancy, nutrition management, postpartum mental adjustment, and infant care; however, prior studies have reported that only 31.7% received prenatal support services due to challenges in communicating in Korean and cultural adaptation [18,20]. Furthermore, pregnancy and childbirth support services are especially urgent for immigrant women given that the spouse’s age significantly affects the outcome of childbirth [19,25]. Immigrant women’s period of adaptation to Korean society and culture should facilitated through stronger societal and policy support, especially before they need prenatal and perinatal support services. Such support will provide the opportunity for all immigrant women to receive pregnancy and childbirth support services.

This study focused on birth data and as such, did not include an analysis of prenatal care or medical records. Therefore, the researchers were limited to using the statistical data presented in this study to analyze immigrant women’s demographic characteristics and their associations with prenatal care and high-risk pregnancy complications. Future researchers should identify the factors influencing the birth of immigrant women and the birth of VLBW children to provide a basis for suggesting interventional plans. Another limitation of the study was not factoring in genetic characteristics, including sex, and physical features such as congenital abnormalities when comparing the weight of infants. In future studies, those factors need to be carefully considered when comparing weights among populations of different races or ethnicities.

In conclusion, childbirth among immigrant women contributes significantly to the quantitative improvement of the Korea’s population structure; however, immigrant women face many challenges to healthy pregnancy and childbirth management, necessitating several qualitative improvements. First, measures should be taken to enhance immigrant women’s adaptation to the Korean medical system and ensure adequate communication with medical personnel. Active support from health care workers and the provision of national-level services are needed. It is also necessary to identify the risk factors of childbirth by nationality and create interventions based on immigrant women’s cultural characteristics. Such improvements will contribute to safer pregnancy and childbirth among immigrant women, which will improve Korea’s population structure.

## Figures and Tables

**Table 1.** Number of live births by nationality of mothers in 2018 (N=15,216)

Rank	Country	Number of live births, n (%)
1	Vietnam	6,411 (42.1)
2	China	3,746 (24.6)
3	Philippines	1,243 (8.2)
4	Cambodia	736 (4.8)
5	Japan	690 (4.5)
6	Thailand	440 (2.9)
7	United States	402 (2.6)
8	Uzbekistan	245 (1.6)
9	Taiwan	222 (1.5)
10	Mongolia	160 (1.1)
11	Canada	122 (0.8)
12	Russia	101 (0.7)
13	Unknown	95 (0.6)
14	Nepal	70 (0.5)
15	Laos	60 (0.4)
16	Indonesia	55 (0.4)
17	Australia	46 (0.3)
18	Others	372 (2.4)

**Table 2.** Birth weight and gestational age among Korean women and immigrant women in 2016-2018

Variable	Cathegories		Korean women, n (%)			Immigrant women, n (%)		
			2016	2017	2018	2016	2017	2018
Birth weight (kg)	<1.5		2,692 (0.7)	2,422 (0.7)	2,312 (0.7)	90 (0.6)	104 (0.7)	126 (0.8)
	1.5–2.49		20,268 (5.2)	18,670 (5.5)	17,092 (5.5)	778 (4.8)	820 (5.3)	703 (4.7)
	2.5–3.99		353,897 (90.8)	309,787 (90.7)	281,137 (90.8)	14,837 (91.4)	13,935 (90.8)	13,521 (91.2)
	≥4.0		12,725 (3.3)	10,504 (3.1)	9,086 (2.9)	530 (3.3)	495 (3.2)	477 (3.2)
	Total		389,582 (100)	341,383 (100)	309,627 (100)	16,235 (100)	15,354 (100)	14,827 (100)
Gestational age (week)	Total	<37	28,331 (7.3)	25,964 (7.6)	24,106 (7.8)	1,058 (6.5)	1,131 (7.4)	1,096 (7.5)
		37–41	360,640 (92.6)	314,980 (92.3)	284,788 (92.1)	15,078 (93.3)	14,115 (92.5)	13,477 (92.3)
		≥42	480 (0.1)	329 (0.1)	412 (0.1)	30 (0.2)	16 (0.1)	27 (0.2)
		Total	389,451 (100)	341,273 (100)	309,306 (100)	16,166 (100)	15,262 (100)	14,600 (100)
	Singleton	<37	18,832 (5.0)	17,452 (5.3)	15,712 (5.3)	786 (5.0)	816 (5.5)	822 (5.8)
		37–41	354,870 (94.8)	310,090 (94.6)	280,003 (94.6)	14,887 (94.8)	13,936 (94.4)	13,317 (94.0)
		≥42	480 (0.1)	329 (0.1)	406 (0.1)	30 (0.2)	16 (0.1)	27 (0.2)
		Total	374,182 (100)	327,871 (100)	296,121 (100)	15,703 (100)	14,768 (100)	14,166 (100)
	Multifetal	<37	9,499 (62.2)	8,512 (63.5)	8,394 (63.7)	272 (58.7)	315 (63.8)	274 (63.1)
		37–41	5,770 (37.8)	4,890 (36.5)	4,785 (36.3)	191 (41.3)	179 (36.2)	160 (36.9)
		≥42	0 (0)	0 (0)	6 (0.0)	0 (0)	0 (0)	0 (0)
		Total	15,269 (100)	13,402 (100)	13,185 (100)	463 (100)	494 (100)	434 (100)

Missing values excluded.

## Supplementary materials

Further details on supplementary materials are presented online (available at https://doi.org/10.4069/kjwhn.2021.03.15).

Supplementary Table 1. Number of live births among Korean women and immigrant women in 2008-2018

Supplementary Table 2. Number of live births by age among Korean women and immigrant women in 2008 and 2018

Supplementary Table 3. Age of Korean women, immigrant women, and their husbands at childbirth and length of marriage at the first childbirth in 2008 and 2018
