# Supplementary material for: Comparison of the number of live births, maternal age at childbirth, and weight of live births between Korean women and immigrant women in 2018
Source: Korean J Women Health Nurs. 2021 Mar 23;27(1):40–8. doi: 10.4069/kjwhn.2021.03.15 (PMC9334167; doi:10.4069/kjwhn.2021.03.15)
Supplement: Supplementary Table 1. — Number of live births among Korean women and immigrant women in 2008-2018 [file kjwhn-2021-03-15-suppl1.pdf]

Supplementary Table 1. Number of live births among Korean women and immigrant women in 2008-2018

Year	Total	Korean women, n (%)	Immigrant women, n (%)			Unknown, n (%)
			Total	Naturalized Koreans	Foreign nationals	
2008	465,892	451,376 (96.9)	11,690 (2.5)	1,562 (0.3)	10,128 (2.2)	2,826 (0.6)
2009	444,849	425,303 (95.6)	17,106 (3.8)	1,907 (0.4)	15,199 (3.4)	2,440 (0.5)
2010	470,171	449,466 (95.6)	17,997 (3.8)	2,352 (0.5)	15,645 (3.3)	2,708 (0.6)
2011	471,265	450,528 (95.6)	19,374 (4.1)	2,954 (0.6)	16,420 (3.5)	1,363 (0.3)
2012	484,550	463,545 (95.7)	19,893 (4.1)	3,024 (0.6)	16,869 (3.5)	1,112 (0.2)
2013	436,455	417,524 (95.7)	18,219 (4.2)	2,886 (0.7)	15,333 (3.5)	712 (0.2)
2014	435,435	416,882 (95.7)	17,895 (4.1)	3,180 (0.7)	14,715 (3.4)	658 (0.2)
2015	438,420	421,569 (96.2)	16,621 (3.8)	3,238 (0.7)	13,383 (3.1)	230 (0.1)
2016	406,243	389,750 (95.9)	16,282 (4.0)	3,264 (0.8)	13,018 (3.2)	211 (0.1)
2017	357,771	342,116 (95.6)	15,485 (4.3)	3,219 (0.9)	12,266 (3.4)	170 (0.0)
2018	326,822	311,418 (95.3)	15,216 (4.7)	3,354 (1.0)	11,862 (3.6)	188 (0.1)

https://doi.org/10.4069/kjwhn.2021.03.15
