# Supplementary material for: Comparison of the number of live births, maternal age at childbirth, and weight of live births between Korean women and immigrant women in 2018
Source: Korean J Women Health Nurs. 2021 Mar 23;27(1):40–8. doi: 10.4069/kjwhn.2021.03.15 (PMC9334167; doi:10.4069/kjwhn.2021.03.15)
Supplement: Supplementary Table 2. — Number of live births by age among Korean women and immigrant women in 2008 and 2018 [file kjwhn-2021-03-15-suppl2.pdf]

Supplementary Table 2. Number of live births by age among Korean women and immigrant women in 2008 and 2018

Age (year)	All, n (%)		Korean women, n (%)		Immigrant women, n (%)	
	2008	2018	2008	2018	2008	2018
≤ 19	2,264 (0.5)	1,300 (0.4)	1,706 (0.4)	1,096 (0.4)	558 (4.8)	204 (1.3)
20–24	27,457 (5.9)	13,312 (4.1)	23,146 (5.1)	10,330 (3.3)	4,311 (36.9)	2,982 (19.6)
25–29	168,423 (36.4)	65,027 (19.9)	164,846 (36.5)	60,300 (19.4)	3,577 (30.6)	4,727 (31.1)
30–34	198,370 (42.8)	143,063 (43.8)	196,290 (43.5)	138,358 (44.4)	2,080 (17.8)	4,705 (30.9)
35–39	59,492 (12.9)	91,101 (27.9)	58,546 (13.0)	88,959 (28.6)	946 (8.1)	2,142 (14.1)
40–44	6,530 (1.4)	12,417 (3.8)	6,331 (1.4)	11,980 (3.8)	199 (1.7)	437 (2.9)
≥ 45	362 (0.1)	407 (0.1)	348 (0.1)	390 (0.1)	14 (0.1)	17 (0.1)
Total	462,898 (100)	326,627 (100)	451,213(100)	311,413 (100)	11,685 (100)	15,214 (100)

Missing values excluded.
