# Supplementary material for: Comparison of the number of live births, maternal age at childbirth, and weight of live births between Korean women and immigrant women in 2018
Source: Korean J Women Health Nurs. 2021 Mar 23;27(1):40–8. doi: 10.4069/kjwhn.2021.03.15 (PMC9334167; doi:10.4069/kjwhn.2021.03.15)
Supplement: Supplementary Table 3. — Age of Korean women, immigrant women, and their husbands at childbirth and length of marriage at the first childbirth in 2008 and 2018 [file kjwhn-2021-03-15-suppl3.pdf]

Supplementary Table 3. Age of Korean women, immigrant women, and their husbands at childbirth in 2008-2018

Variable	Categories	Mean	
		Korean women N (%)	Immigrant women N (%)
Age of woman (year)	First childbirth	31.6	28.2
	Second childbirth	33.2	30.1
	Third or more childbirth	35.0	31.7
	Unknown	32.9	29.7
	All	32.5	29.3
Age of husband (year)	First-born	33.8	39.6
	Second-born	35.4	41.0
	Third or later-born	37.3	42.2
	All	34.7	40.3

Missing values excluded.

https://doi.org/10.4069/kjwhn.2021.03.15
